# PLD2–PI(4,5)P2 interactions in fluid phase membranes: Structural modeling and molecular dynamics simulations

**DOI:** 10.1371/journal.pone.0236201

**Published:** 2020-07-20

**Authors:** Kyungreem Han, Richard W. Pastor, Cristina Fenollar–Ferrer

**Affiliations:** 1 Laboratory of Computational Biology, National Heart, Lung and Blood Institute, National Institutes of Health, Bethesda, Maryland, United States of America; 2 Laboratory of Molecular & Cellular Neurobiology, National Institute of Mental Health, National Institutes of Health, Bethesda, Maryland, United States of America; 3 Laboratory of Molecular Genetics, National Institute on Deafness and other Communication Disorders, Bethesda, Maryland, United States of America; 4 Molecular Biology and Genetics Section, National Institute on Deafness and other Communication Disorders, Bethesda, Maryland, United States of America; University of Lincoln, UNITED KINGDOM

## Abstract

Interaction of phospholipase D2 (PLD2) with phosphatidylinositol (4,5)-bisphosphate (PIP_2_) is regarded as the critical step of numerous physiological processes. Here we build a full-length model of human PLD2 (hPLD2) combining template-based and *ab initio* modeling techniques and use microsecond all-atom molecular dynamics (MD) simulations of the protein in contact with a complex membrane to determine hPLD2-PIP_2_ interactions. MD simulations reveal that the intermolecular interactions preferentially occur between specific PIP_2_ phosphate groups and hPLD2 residues; the most strongly interacting residues are arginine at the pbox consensus sequence (PX) and pleckstrin homology (PH) domain. Interaction networks indicate formation of clusters at the protein-membrane interface consisting of amino acids, PIP_2_, and 1-palmitoyl-2-oleoyl-sn-glycero-3-phosphatidic acid (POPA); the largest cluster was in the PH domain.

## 1. Introduction

Phospholipase D2 (PLD2) is a phospholipid-metabolizing enzyme of the phospholipase superfamily that is involved in numerous intracellular signal transduction pathways that affect a wide variety of physiological processes [1, 2]. Hence, the dysregulation of PLD2 is linked to many pathological conditions, including different types of cancers [3] and neurological disorders such as Alzheimer's and Parkinson's [4]. Human PLD2 (hPLD2) consists of two HKD domains (named HKD1 and HKD2) containing the HKD consensus signature motif HxKxxxxDxxxxxxGSxN [5], a pbox consensus sequence (PX) domain and a pleckstrin homology (PH) domain. HKDs are essential for mediating the hydrolytic activity of PLD2, while the PX and PH domains regulate such activity by interacting with lipids and other proteins [1, 2, 5–9].

The lipid headgroups phosphatidylinositol (PI) bisphosphates including PI(4,5)P_2_ (henceforth referred to as PIP_2_), PI(3,4)P_2_, and PI(3,5)P_2_ are considered to be critical regulators of peripheral and integral membrane proteins [10–15]. In particular, PIP_2_ is essential for the intracellular localization of PLD2 [1, 2, 5, 7–9]. Furthermore, it has recently been suggested that PIP_2_ cluster-PLD2 interaction plays a primary role in the action of general anesthetics (GAs) [16–18]. The mechanism of general anesthesia, ‘a reversible, drug-induced loss of consciousness’, is one of the unsolved problems of neuropharmacology [19–22]. In particular, there is a long-standing debate whether GAs act through non-specific perturbations of lipid bilayers (‘lipid theory’) or directly on specific sites of transmembrane effector proteins (‘protein theory’) [19–28]. The primary target of GAs is the potassium channel TREK-1 (TWIK-related K^+^ channel 1 [29]) and they act by inducing the opening of the channel in a three-step cycle: (1) lipid rafts containing PLD2 are disrupted by the GA releasing PLD2 [1, 16–18]; (2) PLD2 then catalyzes the hydrolysis of phosphatidylcholine (PC) to produce the TREK-1 agonist phosphatidic acid (PA) resulting in the inhibition of the membrane excitation by outwardly rectifying K^+^ current [23, 29–34]; (3) when the GA concentration decreases the PLD2 binding raft reforms, PLD2 is deactivated, and the effect of the anesthesia is dissipated. PIP_2_ not only regulates TREK-1 activity via direct binding [30, 35–37] but also affects the channel activity via modulating PLD2 function [16–18]. In particular, PIP_2_ clustering is critical for PLD2-lipid bilayer interactions in the presence of GAs; the released PLD2 from the lipid rafts can translocate to the PIP_2_ cluster so that the enzyme can be maintained on the lipid bilayer against GA-induced disruption of lipid rafts. In addition, PIP_2_ itself is an activator of the enzyme [1, 34, 38].

While the preceding mechanism proposed is elegant and reconciles the two seemingly opposing lipid and protein theories, a more detailed molecular description of each of the steps is necessary. One of the main limitations in this regard is the lack of structural information on PLD2. For example, while it has been determined experimentally that the PH domain binds PIP_2_, the interaction of the PX and HKD domains with the membrane surface is less clear. Likewise, the effect of mutations of PLD2 is difficult to interpret without the structural information on the PIP_2_ binding mode.

In this paper, we build a full-length model of human PLD2 (hPLD2) combining template-based and *ab initio* modeling techniques. The resulting model is then used to investigate the interaction mode of hPLD2 with PIP_2_ using all-atom molecular dynamics (MD) simulations including explicit fluid phase membranes with high and low concentrations of PIP_2_. The newly determined structure and protein/membrane MD simulations allow identification of binding residues/phosphate groups of PIP_2_ at the hPLD2-membrane interfaces, and evaluation of PIP_2_ clustering at the binding sites.

By way of outline, Section 2 (Methods) describes the homology modeling of hPLD2 (2.1), the molecular docking (2.2), MD simulations (2.3), and network generation (2.4). Section 3 (Results) begins with the examination of hPLD2 model (3.1), followed by the analysis of hPLD2-PIP_2_ interactions (3.2). Section 4 combines the Discussion and Conclusions.

## 2. Methods

### 2.1. Homology modeling of PLD2

#### 2.1.1. Identification of templates

Template-based molecular modeling of a query sequence relies mainly on the algorithm used to identify possible structures that have the same or similar fold. Fold recognition methods such as HHpred [39, 40] that use hidden Markov model (HMM) profiles instead of single sequences are the most sensitive identifying suitable templates, especially in cases where template and query sequence share very low sequence identity [41]. In this work, HHpred server [39, 40] was used to identify possible templates for hPLD2 using the amino acid sequence of the protein as input. The HMM profile of PLD2 was obtained after three iterations of HHblits [41] using uniclust30 sequence database with an E-value inclusion threshold of 10^−3^ and a minimum sequence coverage of 20%. The PLD2 profile containing the 250 closest homologs was then scanned against the database of the HMM profiles for each of the Protein Data Bank (PDB) crystallographic structures (PDB_mmCIF70 database dated April 10th, 2019). The crystallographic structures of the PX-PH domain tandem of Rem3 from *Saccharomyces cerevisiae* (PDBid: 6fsf, resolution 2.2 Å [42]) and phosphatidylserine synthase from *Haemophilus influenzae* Rd KW20 (PDBid: 3hsi, resolution 2.2 Å) were selected as suitable templates for PLD2 segments containing residues 64–314 (PX and PH domains, chain A, template 6fsf) and 331–803 (HKD1 and HKD2 domains, chain B, template 3hsi). Both templates have maximum sequence coverage, highest sequence identity percentage (13% and 15% for 6fsf and 3hsi respectively) and best correspondence between secondary structural elements in their corresponding segments.

After the present structure was determined from 3hsi a HHpred run dated February 2020 also found the structure of the Phospholipase D alpha 1 from *Arabidopsis thaliana* (PDB id: 6kz9 [43]) as a possible template for chain B of PLD2. The structural superimposition of the segments in both templates found to have a similar overall fold, mainly differing in the configuration of several loops (S1 Fig in S1 File). These differences would most probably be neutralized during the Molecular Dynamics simulations performed on the final model, where the loops undergo major rearrangements due mainly to the presence of a membrane and solution. This indicates that both templates are suitable for the modeling of chain B of PLD2 through 3hsi is used here.

#### 2.1.2. Modeling of hPLD2

**Fig 1:**
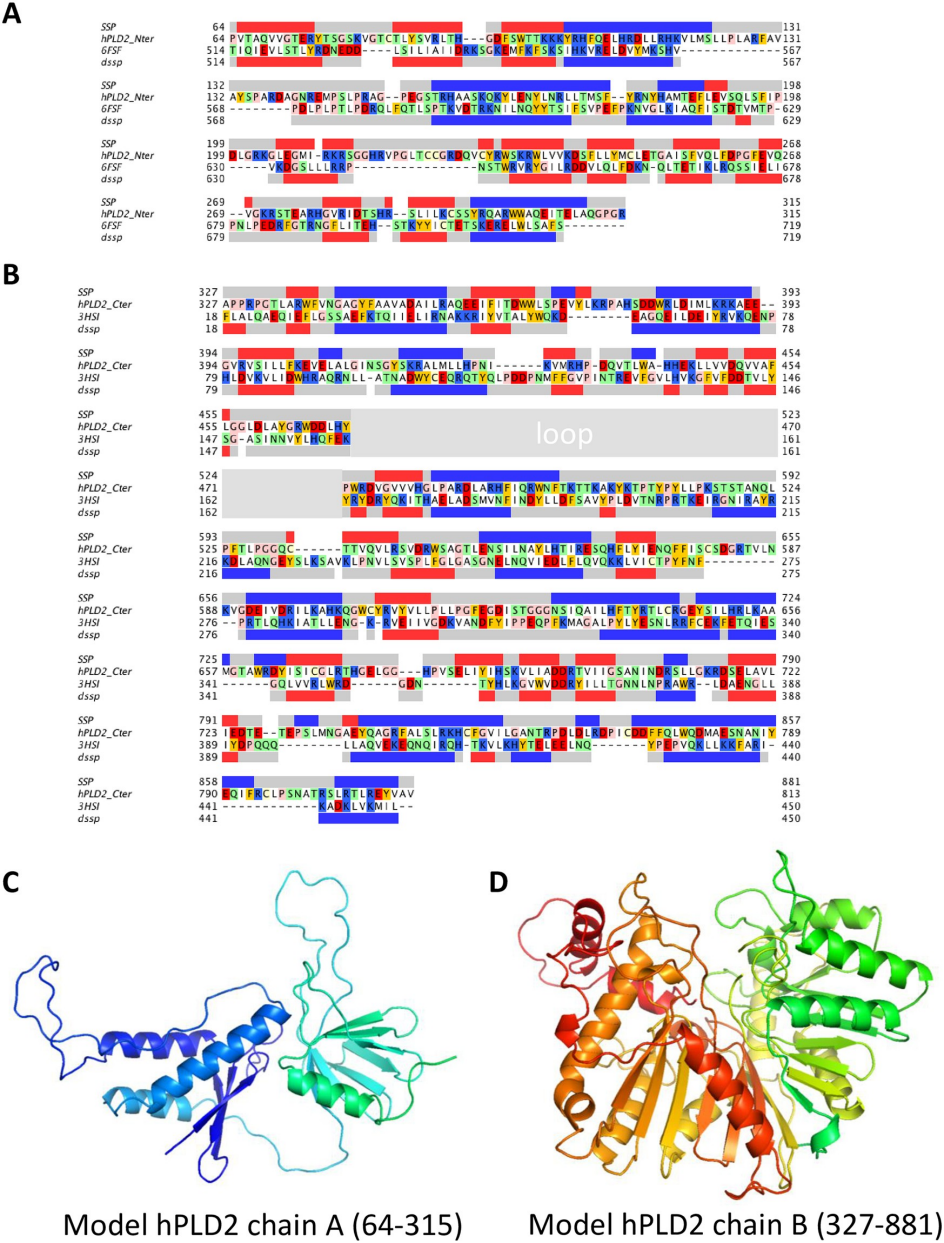
Structural modeling chains A and B of human PLD2. Sequence alignments used to model chains A (residues 64–315) and B (residues 327–881) of human PLD2 (PDB ids: 6fsf and 3hsi). The secondary structure prediction (SSP) for hPLD2 is listed at the top each alignment by grey (coil), red (strand) and blue (helix) bars; the secondary structure from the structure of each template (dssp) is below the sequence. The loop in chain B for which there is no template is indicated with a gray bar. Residues in the sequence are highlighted as follows: basic (blue), acidic (red), polar (green), apolar (white) aromatic (orange), glycine and proline (pink). The final models for chains A and B are shown in C and D in ribbon style.

The initial alignments obtained with HHpred for chains A and B (S2A Fig in S1 File) were subjected to several refinement iterations using the conservation scores calculated with ConSurf server [44] to position the most conserved residues in the internal regions of the protein, removing gaps within secondary structural elements to preserve as much of the protein fold as possible and using local ProQ2 score [45] to quantify if the 3D disposition of the residues in a given segment was in agreement with their sequence conservation and secondary structure prediction. The final alignments for chains A and B (Fig 1A and 1B) were used to generate a set of 2000 models per chain using MODELLER software [46]. The final models were that with the best MODELLER probability distribution function (molPDF) score (Fig 1C and 1D).

**Fig 2:**
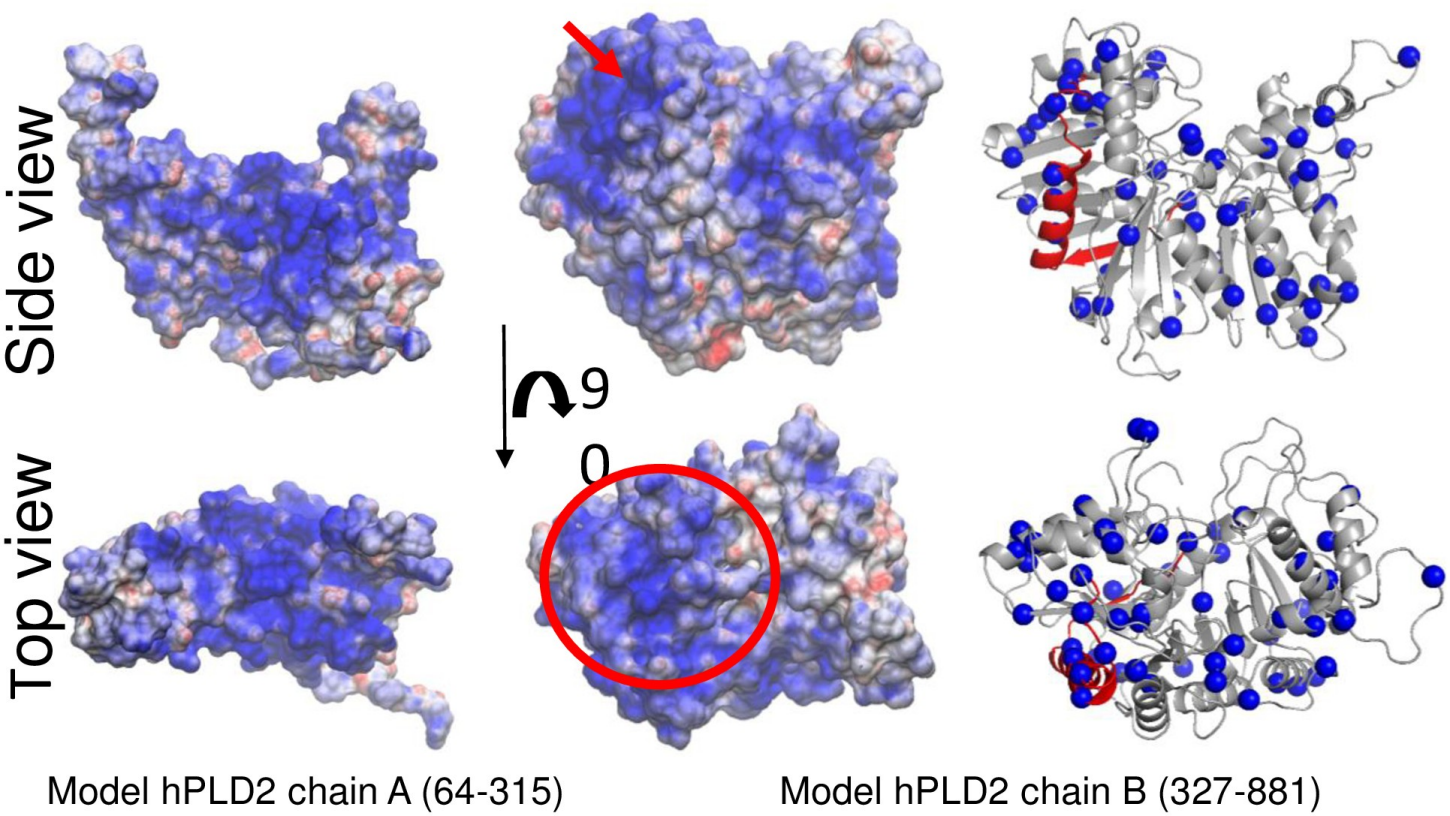
Electrostatic potential analysis. Electrostatic potential mapped on the surface representation of chains A and B of hPLD2. The cluster of positive residues proposed by Sciorra et al [48] to be part of a PIP_2_ binding site in chain B is highlighted with a red arrow (side view) and circle (top view). The segment is rendered in red in the ribbon representation of chain B model and the Cα of any lysine or arginine are rendered as blue spheres (third column).

The residues that were not modeled using the template-based approach were built via *ab initio* folding technique onto the modeled chains using I-TASSER server [47]. The initial relative orientation of the chains positioned the surfaces containing the last residue of chain A and the first of chain B facing each other. In addition, the clusters of basic residues in both chains were positioned on the same plane to maximize their interaction with PIP_2_ in the membrane (Fig 2 and S3 Fig in S1 File). The corresponding file containing the models of chain A and B was used input in I-TASSER server [47], where the residues missing were built using the default parameters of the server. The final full-length model of hPLD2 was the representative of the most populated cluster with the best C-score. Throughout the paper, residues 1 to 325 (including PX and PH domains) and 326 to 933 (HKDs) are referred to as ‘N-term’ and ‘C-term’, respectively. (These are close to, but not identical, to chains A and B discussed above.)

### 2.2. Molecular docking

**Fig 3:**
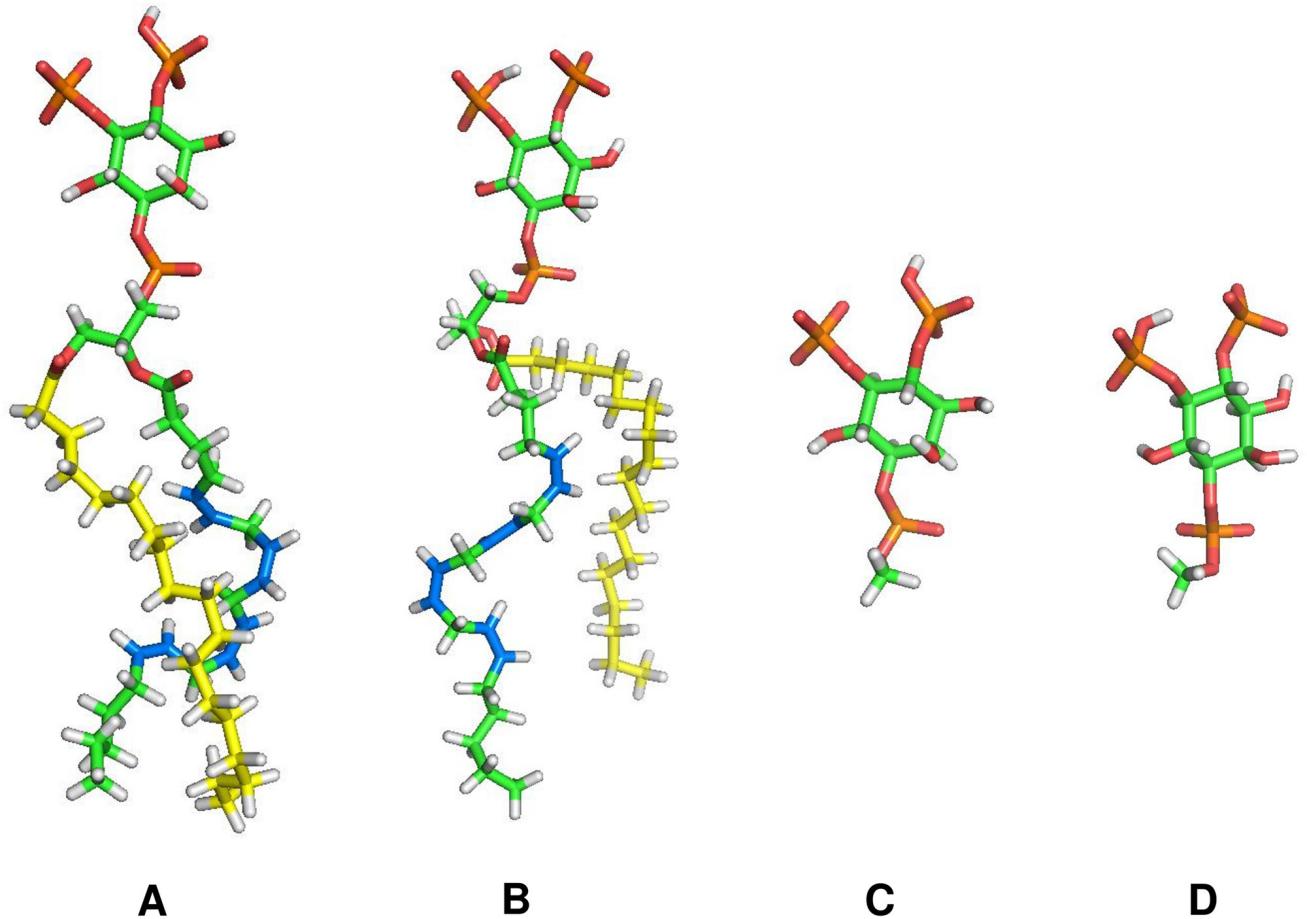
PIP_2_ structures used in MD simulations (A and B) and docking calculations (C and D). **A** PIP_2_ headgroup singly-protonated at 4-phosphate on inositol ring and with stearic (18:0) and arachidonic (20:4) acid chains; **B** preceding PIP_2_ singly-protonated at 5-phosphate; **C** headgroup of **A**; **D** headgroup of **B**. Coloring: hydrogen, white; oxygen, red; phosphate, orange; saturated carbon on steric acid chain, yellow; unsaturated carbon on arachidonic acid chain, blue; all other saturated carbons, green.

Searching for the PIP_2_ binding sites was performed via molecular docking using an AutoDock Vina program [49] with the Amber force field [50]. The tail-truncated structures of the two PIP_2_ models were used in the calculations (Fig 3C and 3D). The calculations were conducted on search spaces defined by cubic boxes of 30 Å edge; the randomly chosen 2×10^3^ initial binding conformations were tested for each search space for enhanced sampling.

### 2.3. MD simulations

The protonation states of hPLD2 model were titrated using PROPKA 3.1 [51], missing atoms were added, and the hydrogen bond networks were optimized using PDB2PQR 2.1 [52]. The protein was incorporated into the bilayers using CHARMM-GUI [53]. The stability of the protein model was tested using a 400 ns unbiased simulation in ~150 mM KCl solutions. As shown in S4 Fig in S1 File the time evolution of the root-mean-square deviation (RMSD) of C_α_ of the entire protein and respective domains indicated that the protein was equilibrated within ~100 ns and remained stable for the following 300 ns of simulation. While the RMSD of domains (i.e., PX, PH, HDK1, and HDK2) showed a small-to-moderate increase with respect to the homology model, that of whole protein increased rapidly due to the flexible linker between the N and C-terminal regions. All the secondary structural elements were maintained during the simulation.

Table 1 lists the components of the 2 bilayer systems (i.e., 1 and 10% PIP_2_ bilayers); they are asymmetric and approximate the composition of fluid phase cell membranes. The lipid models in the Chemistry at HARvard Molecular Mechanics (CHARMM) [54] were used; in particular, the two PIP_2_ models each having stearic (18:0) and arachidonic (20:4) acid chains with singly-protonated at 4- or 5-phosphate on the inositol ring, respectively (Fig 3A and 3B), were randomly distributed on the inner leaflet in a 1:1 ratio. The systems were fully hydrated with 144 TIP3P water [55] per lipid and KCl was added to achieve a ~150 mM bulk concentration.

**Table 1:** Lipid component numbers in MD systems.

System^a^	Leaflet	Lipids^b^						
		PIP_2_^c^	Chol.	POPC	POPE	POPA	PSM	Total
**1% PIP**_**2**_	upper	0	210	240	30	0	120	600
	lower	6	210	144	174	6	60	600
**10% PIP**_**2**_	upper	0	210	240	30	0	120	600
	lower	60	180	90	150	60	60	600

^a^Each system was simulated for three replicas with different random seed for initial velocities. The 100% PIP_2_ systems are dealt with in the main text, while the 1% PIP_2_ systems are discussed in the SI.

^b^PIP_2_, phosphatidylinositol (4,5)-bisphosphate; Chol., cholesterol; POPC, 1-palmitoyl-2-oleoyl-sn-glycero-3-phosphocholin; POPE, 1-Palmitoyl-2-oleoyl-sn-glycero-3-phosphoethanolamine; POPA, 1-palmitoyl-2-oleoyl-sn-glycero-3-phosphatidic acid; PSM, palmitoylsphingomyelin.

^c^A 1:1 mixture of PIP_2_ models of singly-protonated at 4- or 5-phosphate on inositol ring was used.

The systems were minimized using the steepest descent algorithm and heated to 293.15 K over 40 ps, and then simulated to 500 ns under constant number, pressure, and temperature (293.15 K) using the Nosé–Hoover thermostat [56]. The integration time step equals 1 fs (for additional accuracy [57]), and coordinate sets were saved every 5 ps. Electrostatics were evaluated using particle–mesh Ewald (PME) with ca. one grid point per angstrom (Å), a sixth-order spline interpolation for the complementary error function, a *κ* value of 0.32, and a 12 Å real space cutoff. The van der Waals term used a standard 6–12 LJ form, with force–switched truncation over the range 8–12 Å. The SHAKE constraint method [58] was applied to all covalent bonds to hydrogen, with the default tolerance (1.0 × 10^−10^ Å). The OpenMM [59] program was used for the MD simulations. The CHARMM C36 lipid [60] and protein [61–63] force fields were used. Three replicas (R1, R2, R3) were simulated for each system with different random seeds for initial velocities. R1 of the 10% PIP_2_ systems was extended to 3 μs using the ANTON 2 supercomputer [64] with a 2.5 fs integration time step, with coordinate frames saved at 240 ps intervals. The systems contained 580,149 (1% PIP_2_) and 582,319 (10% PIP_2_) atoms. Trajectories were generated at rates of ~4.5 ns per day by OpenMM on an NVIDIA V100 GPU and ~3.4 μs per day by Anton 2.

### 2.4. Network generation

The hPLD2-lipid bilayer interaction networks were constructed based on the MD trajectories similarly to the previous study [65, 66]: each residue of hPLD2 was taken to be connected to PIP_2_ or POPA (node or vertice) by a link (or edge) if the distance between their heavy atoms was less than the distance threshold of 3.5 Å. The heavy atoms separated in the primary cell that interacts with their images within the distance threshold are taken to be connected. The directionality of the links was not considered in the network (i.e., undirected network).

The creation of the connectivity matrix from the MD trajectory was carried out using tailored CHARMM [54] and Python scripts. Visualization of networks was aided by open-source Java application, Cytoscape (version 3.7.2) [67].

## 3. Results

### 3.1. Full-length model of *human* PLD2

**Fig 4:**
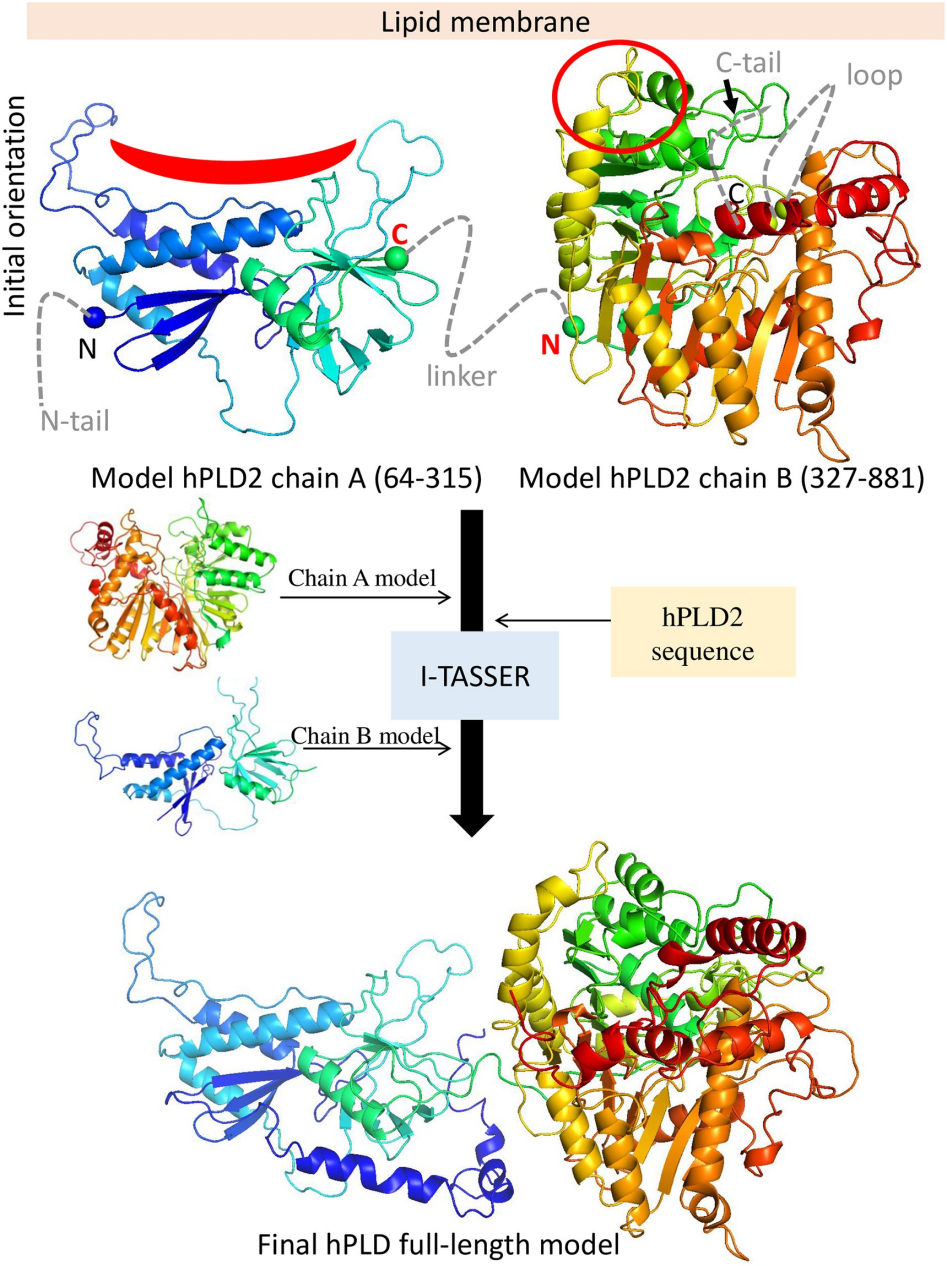
Full-length modeling of hPLD2. The models of chains A and B are oriented relative to a membrane (tan rectangular slab) using the electrostatic potential and putative PIP_2_ binding residues (regions highlighted in red) as guides. Residues 315 (the C-terminal of chain A) and 327 (the N-terminal of chain B) are connected by a 12 residue linker. The residues not modeled in the template-based step are indicated as dashed lines and were subsequently modeled using *ab initio* folding in I-TASSER server. The initial chain A and B models and the amino acid sequence of hPLD2 were used as inputs during the *ab initio* step and the resulting full-length model is shown in the lower panel.

The computational protocol used in this work to model hPLD2 was divided into two consecutive but distinct steps: a) template-based modeling followed by b) *ab initio* modeling (flowchart of the modeling protocol shown in Fig 4).

The crystallographic structure of PX-PH domain tandem of Rem3 from *Saccharomyces cerevisiae* (PDBid: 6fsf, resolution 2.2 Å [42]) and that of phosphatidylserine synthase from *Haemophilus influenzae* Rd KW20 (PDBid: 3hsi, resolution 2.2 Å) were identified as the most suitable templates for PLD2 using HHpred server [39, 40] (run dated April-2019). Each of the templates covered different and non-overlapping segments of PLD2. 6fsf was similar to residues located on the first half of PLD2 (64–314) and that included the PX and PH domains (referred as chain A), while 3hsi was similar to those located on the second half of PLD2 (331–803) and that included the HKD1 and HKD2 domains (referred as chain B). HHpred is a HMM-based profile algorithm that ensures a higher sensitivity in template detection; being able to detect templates that share very low sequence identity but the same fold with the protein of interest. This is the case of templates 6fsf and 3hsi identified in this study, as despite the lower sequence identity shared with PLD2 (13% and 15% for 6fsf and 3hsi respectively) they have very good correspondence between secondary structural elements (Fig 1A and 1B) and together cover 75% of hPLD2 sequence.

The refined sequence alignments between the two PLD2 segments and their correspondent template were used to generate the models of both moieties (Fig 1C and 1D). In both models, the majority of the most conserved residues are not exposed to the solvent but rather forming part of the structure packing (S2B Fig in S1 File). The stereochemistry of both models was evaluated using PROCHECK analysis [68] showing a maximum of three residues in the disallowed regions of the Ramachandran plot. These residues are all located in loops indicating that in overall the chain A and chain B models obtained have good stereochemistry.

In the second step of the protocol we used *ab initio* folding to model the residues for which no template was identified; these residues were folded onto a single structure containing the models of chains A and B. These two chains are linked by only 12 residues. This short linker raised the question of whether these two segments could be sufficiently close to establish interactions with each other, assumed to be the case in a previous study by Mahankali [69]. We performed a coevolutionary sequence analysis on PLD2 to identify coevolved residue pairs, which frequently correspond to physical contacts into a protein’s 3D-structure. We used two different protocols implemented in EVFold and GREMLIN servers [70, 71] but neither of them identified coevolved pairs between residues of chains A and B. The lack of coevolved residue pairs between chains indicated that most probably they do not interact with each other.

The full-length model of PLD2 was that from the most populated cluster with the highest score obtained using the *ab initio* folding protocol implemented in I-TASSER server [47]. The models obtained in the previous step (chains A and B) were used as input and their relative orientation was that where the largest clusters of positively charged residues were positioned in the same plane to maximize interactions with PIP_2_ in the membrane (Fig 2 and S3 Fig in S1 File). In addition, the plane containing the last residue of chain A was facing that containing the first of chain B (Fig 4).

### 3.2. PLD2-PIP_2_ interactions

**Fig 5:**
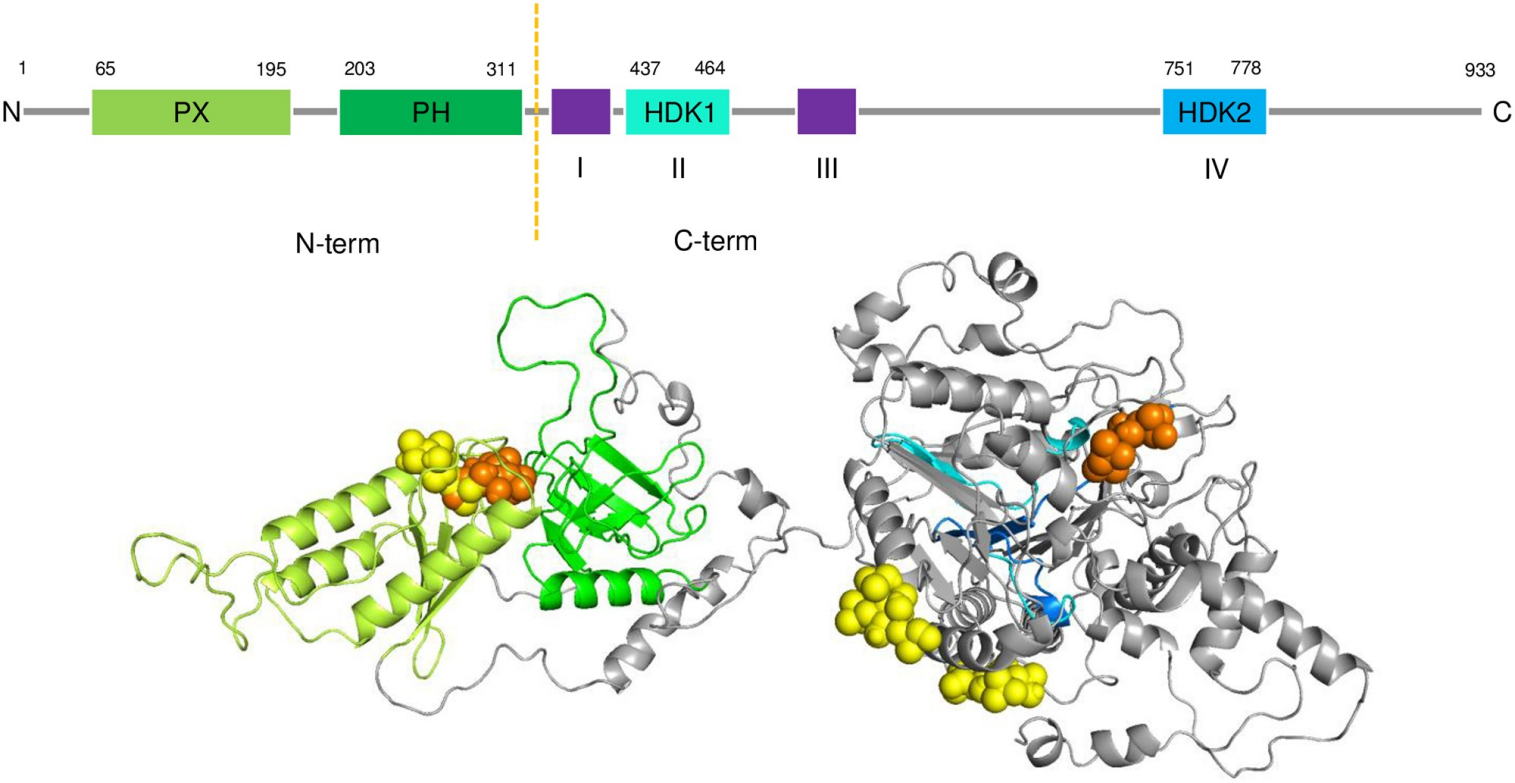
Docking of tail-truncated PIP_2_ on the hPLD2 model. (top) The modular architecture of hPLD2 gene: domains PX (pbox consensus sequence) and PH (pleckstrin homology) are located in N-term (residues from 1 to 325), while C-term (326 to 933) contains four evolutionary conserved sequences (I−IV), where II and IV are HDK motifs. (bottom) Five most stable binding poses of tail-truncated PIP_2_ (singly-protonated at P4) shown as orange/yellow ball representations. The orange represents the most stable pose for each segment (N-term or C-term). Coloring of named protein regions; PX, light green; PH, green; HDK1, cyan; HDK2, blue.

Fig 5 highlights the five most stable binding poses of PIP_2_ headgroup (singly-protonated at P4) on the hPLD2 model obtained from docking calculations. The resulting binding free energies (based on Amber force field [50]) are -8.5 and -6.1 kcal/mol in N-term (marked orange and yellow, respectively), and -8.2 (orange) and ~ -5.8 (two yellow ones) kcal/mol in C-term. The binding poses and free energies for the PIP_2_ headgroup singly-protonated at P5 show no differences.

This docking information was used to determine the starting position of hPLD2 for the MD simulations—the hPLD2 model was placed facing the PIP_2_ binding residues toward the inner leaflet surface. The initial position of hPLD2 was identical for all six systems (three replicas for each of 1% and 10% PIP_2_ systems) as shown in S5 Fig in S1 File.

**Fig 6:**
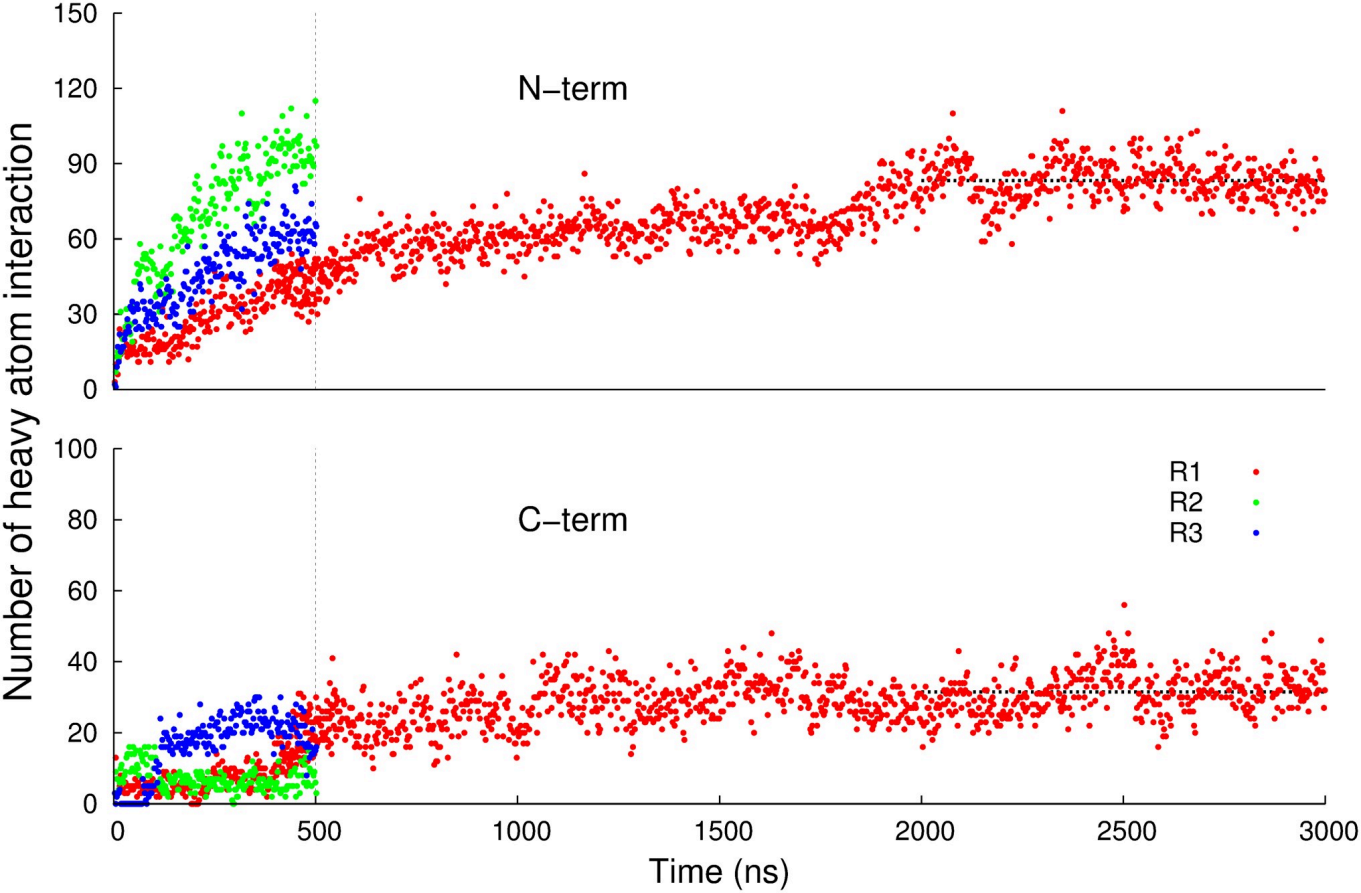
Time profiles of the numbers of contacts between PIP_2_ and PLD2 residues within 3.5 Å. Data were obtained from the three replicas of the 10% system (red, green, and blue for R1, R2, and R3 in S5 Fig in S1 File, respectively). R1 has been extended to 3μs, while R2 and R3 were simulated for 500 ns. It was calculated for the two segments of hPLD2 separately: ‘N-term’ in the first row and ‘C-term’ in the second row. The vertical dotted line indicates the border between N-term and C-term. The horizontal lines are average numbers for 2–3 μs: 83.3 ± 7.9 (standard deviation) for N-term and 31.5 ± 5.9 for C-term.

Fig 6 displays the time evolution of the numbers of contacts between the heavy atoms of hPLD2 and PIP_2_ within 3.5 Å for 10% PIP_2_ systems (R1 –R3). It is clear that the number of N-term contacts with PIP_2_ is about 2.5 times more than that of C-term. The average contact numbers during the last microsecond (i.e., 2–3 μs) for R1 is 83.3 ± 7.9 (standard deviation) for N-term and 31.5 ± 5.9 for C-term (horizontal dotted lines in Fig 6). The time profiles for another lipid of interest in the presence of GAs, 1-palmitoyl-2-oleoyl-sn-glycero-3-phosphatidic acid (POPA) followed the trend though the contact numbers are substantially less than those of PIP_2_ (S6 Fig in S1 File) even though the numbers of POPA and PIP_2_ are the same (Table 1).

**Fig 7:**
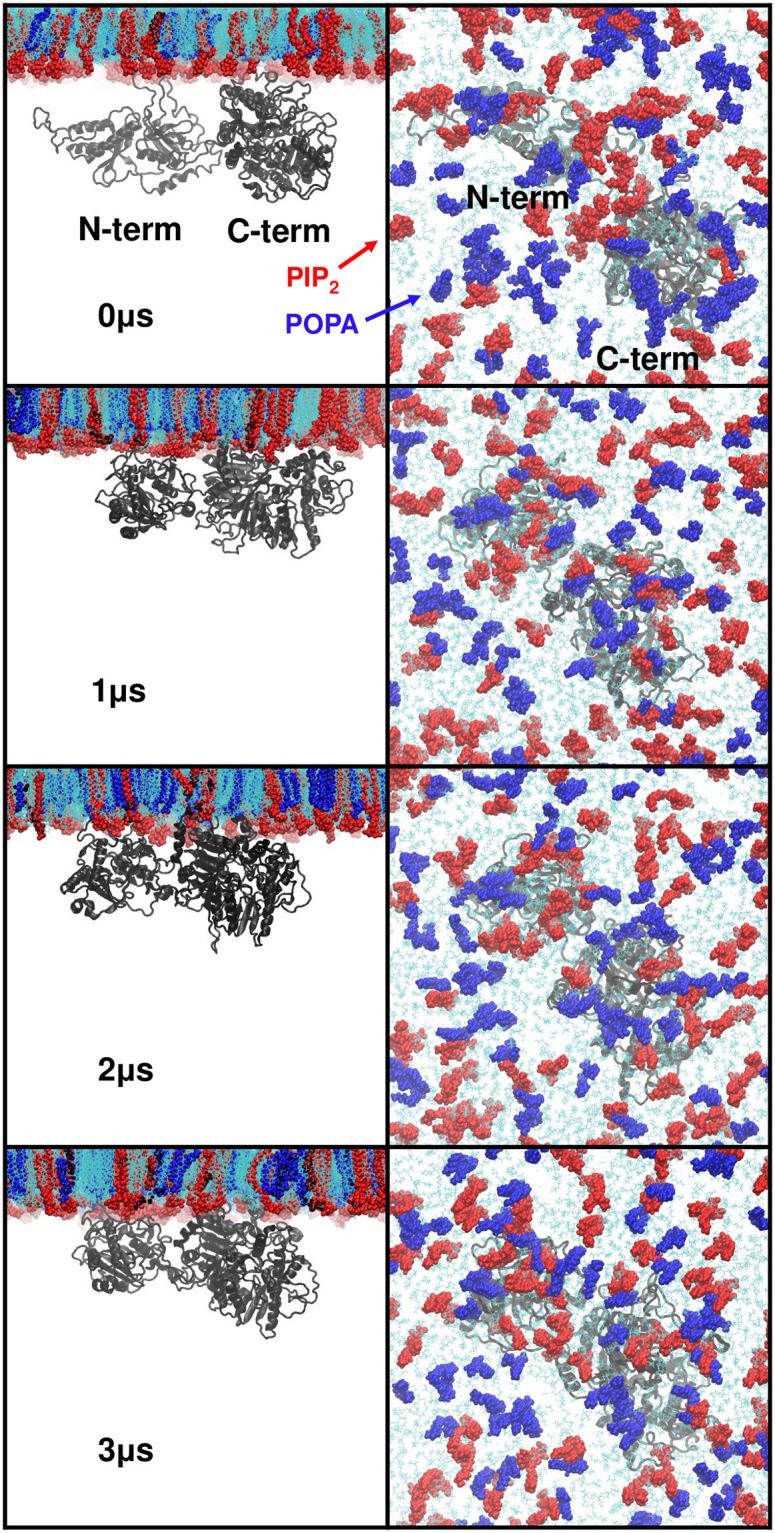
Snapshots of 10% PIP_2_ bilayer system (R1). (left) side view at 0, 1, 2, and 3μs and (right) corresponding top view. Coloring is as follows: PIP_2_, red; POPA, blue; other lipids, cyan; hPLD, gray.

Snapshots at 1μs intervals of the 3μs simulation of R1 are presented in Fig 7, where it is clear that the protein bound more tightly to the membrane as the simulation proceeded. In agreement with the time profiles of hPLD2-PIP_2_ interactions (Fig 6**)**, after 2μs of the simulation, the bindings of both N-term and C-term have converged. On average, 18 (of 60) PIP_2_ interacted with the protein residues during 2–3 μs. Rearrangements of POPA toward hPLD2 residues were less pronounced, with only 6 interacting with the protein in the 2–3 μs interval (Fig 7). There were no protein-lipid interactions at the initial time point of the simulation (Fig 6). There are 60 copies each of these lipids in the leaflet (Table 1). The results here then indicate that 30% of the PIP_2_ and 10% of POPA interact with the protein; i.e., PIP_2_ interacts more strongly with hPLD2 than POPA. POPA might be repelled by PIP_2_ from the protein-bilayer interface though some instances are retained via specific interactions with well-matched residue partners.

The reduction of PIP_2_ concentration from 10% to 1% significantly lowered the number of hPLD2-bilayer interactions. Although the initial positions of hPLD2 for three replicas of 1% PIP_2_ systems were the same as those of 10% PIP_2_ systems, the average protein-membrane separation increased over the 500ns simulations (S5 Fig in S1 File). This reflects the 10-fold reduction in negatively charged lipids and concomitant decrease of protein-membrane electrostatic attraction.

**Fig 8:**
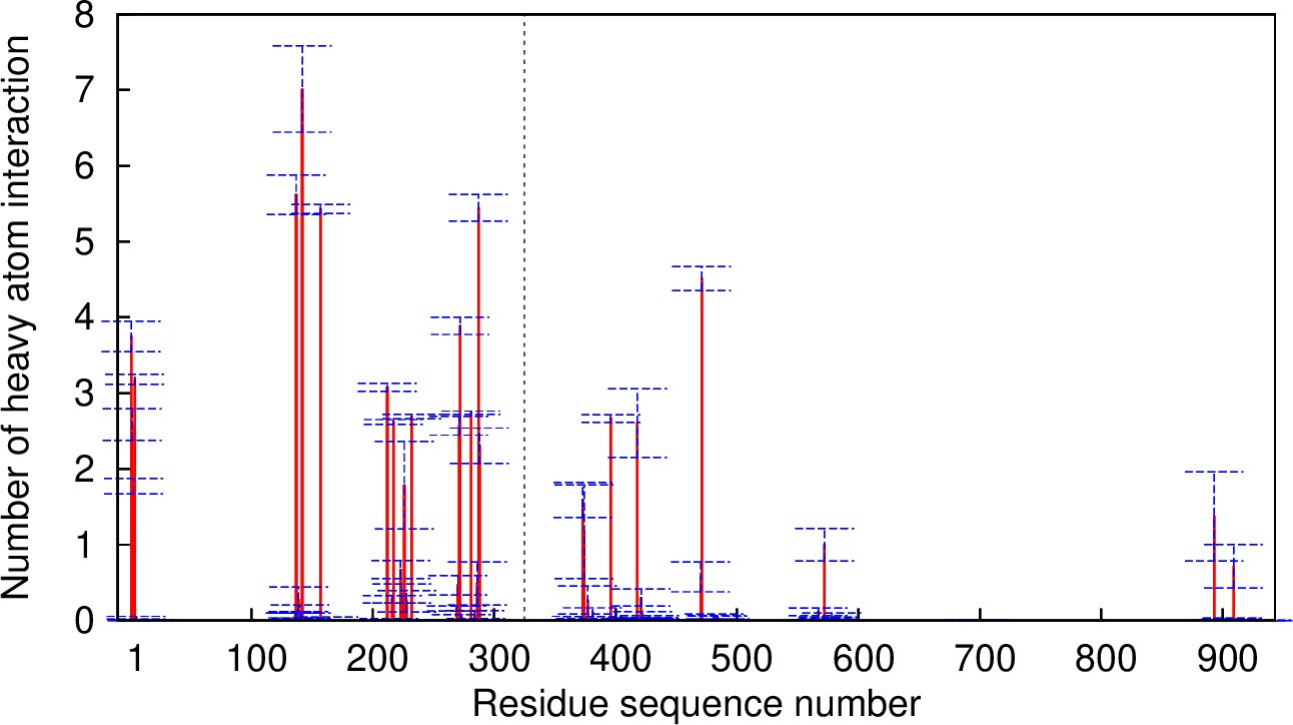
The numbers of contacts between heavy atoms of PIP_2_ and PLD2 residues within 3.5 Å. Data were obtained from the last 1μs trajectories of the replica R1 of the 10% system, dividing into 5 blocks of 200ns to estimate the average and standard error (SE). The vertical dotted line indicates the border between N-term and C-term.

To begin a quantitative analysis of hPLD2-PIP_2_ interactions in molecular detail, the average number of contacts for each hPLD2 residue during 2–3μs was calculated (Fig 8).

The basic amino acid R142 is the strongest binding site for PIP_2_, followed by fairly strong binding at R137, R287, R157, R471, and R272, and then relatively weak binding other arginines. Arginine preferentially binds to the non-protonated phosphate groups of PIP_2_ (P4^np^ and P5^np^, attached to C4 and C5 carbon of the inositol ring, respectively). Those with a strong binding affinity (i.e., the contact number > 2) are located at PX and PH domains of N-term except R471, R396, and R418 (Table 2). Other amino acids (e.g., M1, T4, T2, and S288) also can strongly interact with the phosphate groups other than P4^np^ and P5^np^.

**Table 2:** PIP_2_ binding residues.

Ranking	Residue	^b^Domain	^a^Average contacts with heavy atoms of PIP_2_ (mean ±S.E.)	%Binding lifetime (Number of PIP_2_ partners)	Binding site ratio (%)					
					P1^np^	P4^p^	P5^p^	P4^np^	P5^np^	other
1	R142	N-PX	7.012±0.570	99.8(2)	0.0	11.7	1.1	31.3	53.7	2.2
2	R137	N-PX	5.619±0.259	95.4(2)	0.0	0.7	15.2	74.8	2.4	6.8
3	R287	N-PH	5.443±0.175	100.0(3)	0.0	9.9	17.5	36.9	27.2	8.4
4	R157	N-PX	5.431±0.059	99.8(1)	0.0	14.0	0.0	0.0	76.3	9.7
5	R471	C	4.511±0.157	100.0(2)	0.0	0.0	21.7	68.8	0.0	9.5
6	R272	N-PH	3.889±0.115	100.0(3)	0.0	0.0	9.9	89.9	0.0	0.3
7	M1	N	3.749±0.201	100.0(3)	35.4	0.0	1.9	0.0	60.1	2.6
8	T4	N	3.183±0.064	99.5(1)	0.0	0.0	99.9	0.0	0.0	0.1
9	R212	N-PH	3.080±0.052	99.8(2)	0.0	24.7	0.0	0.0	69.0	6.3
10	R281	N-PH	2.740±0.022	99.8(1)	0.0	18.0	0.0	0.0	82.0	0.0
11	R232	N-PH	2.689±0.029	100.0(1)	0.0	0.0	5.0	95.0	0.0	0.0
12	R396	C	2.665±0.051	98.8(1)	0.0	0.0	6.1	90.7	0.0	3.2
13	R217	N-PH	2.619±0.031	99.0(1)	0.0	0.0	0.0	99.8	0.0	0.2
14	R418	C	2.605±0.453	89.6(1)	11.6	0.0	0.0	0.0	70.0	18.4
15	T2	N	2.585±0.208	94.0(4)	0.1	0.1	29.7	0.0	70.0	0.1
16	K271	N-PH	2.571±0.125	100.0(2)	0.1	0.0	28.2	71.2	0.0	0.5
17	S288	N-PH	2.306±0.232	85.5(1)	0.0	96.9	0.0	0.0	3.1	0.0
18	R226	N-PH	1.783±0.578	66.3(1)	0.0	4.5	0.0	0.0	95.1	4.3
19	A3	N	1.773±0.101	99.8(3)	5.2	0.0	85.6	0.0	0.0	9.2
20	K373	C	1.590±0.232	72.0(3)	0.3	4.5	12.5	31.8	41.0	9.9
21	R893	C	1.373±0.586	51.1(3)	0.0	12.0	0.0	1.4	86.6	0.0
22	R374	C	1.171±0.615	44.1(3)	0.0	0.0	25.8	73.7	0.3	0.3
23	K572	C	1.000±0.212	57.6(1)	0.0	0.0	34.8	63.8	0.0	1.4

^a^Only residues having the average contacts ≥ 1 are displayed.

^b^N and C denote N-term and C-term and PX and PH the pbox consensus sequence and the pleckstrin homology domain, respectively. P1, P4, and P5 indicate heavy atoms of the phosphate group attached to C1, C4 and C5 carbon of the inositol ring and the superscript ‘p’ and ‘np’ denotes protonated and non-protonated phosphate group, respectively, ‘other’ the binding sites other than P1, P4, and P5. %Binding lifetime denotes the fraction of time that a certain residue bound to neighboring PIP_2_ which are identified in parentheses. Data were obtained from the last 1μs trajectories of the replica R1 of the 10% system.

Unlike the interactions with PIP_2_, a relatively small number of residues significantly interact with POPA (S7 Fig and S1 Table in S1 File); only 9 residues have the contact number > 1. Among them, 3 residues (i.e., R217, R226, and R232) interact with both POPA and PIP_2_.

**Fig 9:**
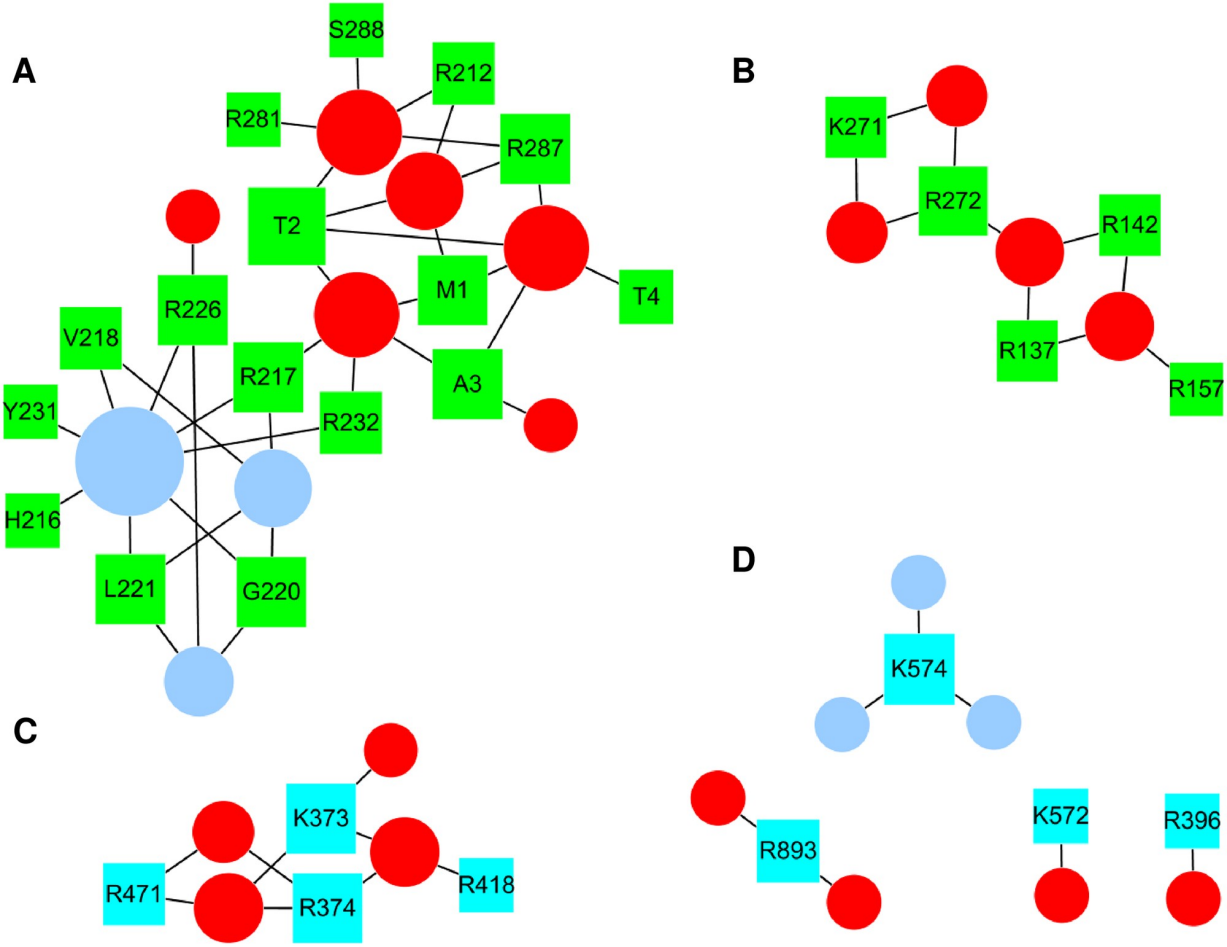
Network representation of hPLD2-lipid bilayer interaction networks. The networks were created based on the average connectivity during 2–3 μs of R1 of the 10% PIP_2_ system (not every link is present at every time point). Green and cyan rectangles denote hPLD2 residues in N-term and C-term, respectively; red circles are PIP_2_, and light blue circles are POPA. The size of the rectangle/circle corresponds to the degree (i.e., the number of links incident to the node). (a) The largest residue cluster (mostly in the PH domain) mediated by PIP_2_ and POPA, (b) A cluster of 5 residues mostly in the PX domain linked by PIP_2_, (c) A cluster of 4 residues linked by POPA in C-term, and (d) monomers in C-term, mediated by either PIP_2_ or POPA.

Fig 9 displays hPLD2-lipid bilayer interaction networks based on the average interactions during 2–3μs. These networks include all the residues listed in Table 2 (for PIP_2_ interaction) and S1 Table in S1 File (for POPA) as nodes, of which the average contacts with PIP_2_ or POPA are greater than 1. The largest cluster was in the PH domain, primarily driven by the interactions of arginine with PIP_2_ and POPA (Fig 9A). Clustering is less evident in C-term (Fig 9C and 9D).

**Fig 10:**
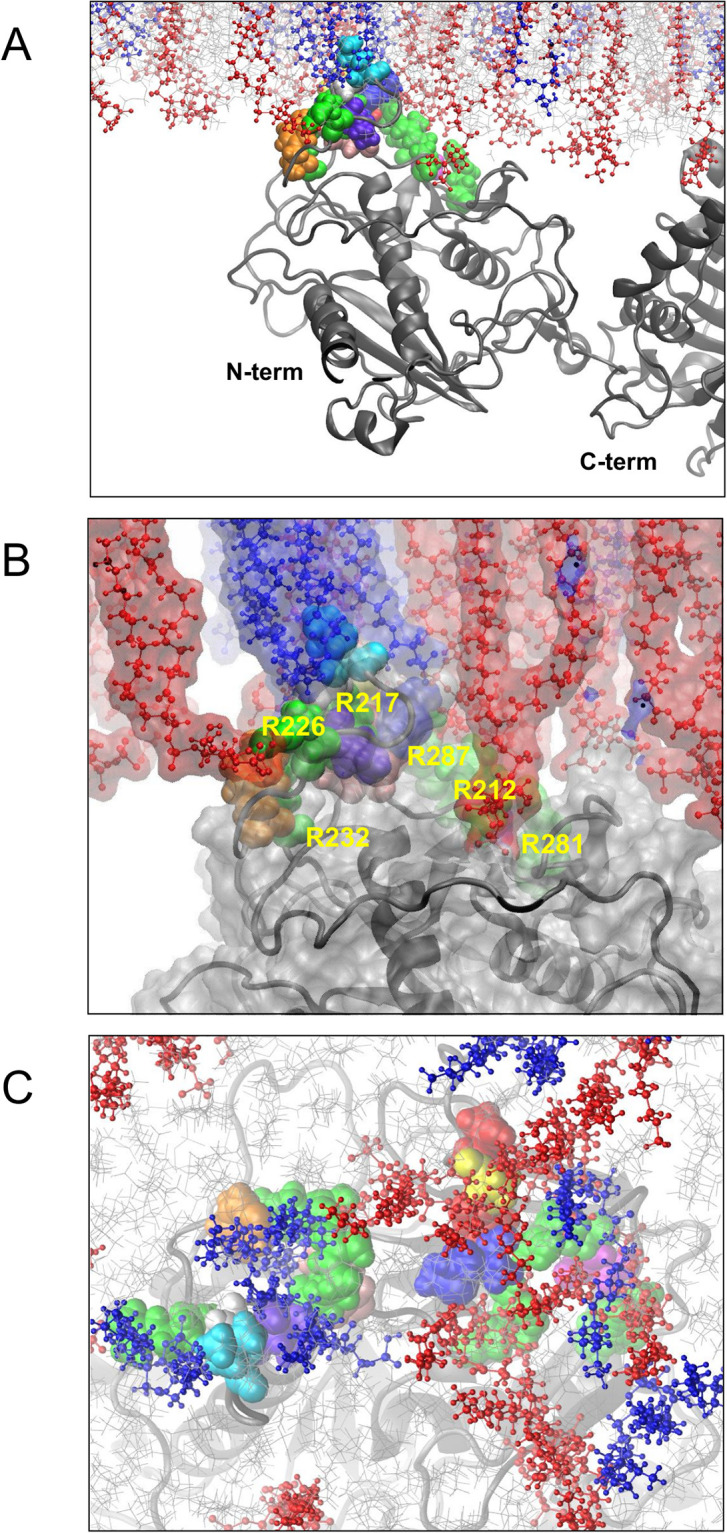
Molecular image of the largest cluster on the PH domain at 3μs. **A** side view of protein and membrane; **B** side view of the molecular surface plot of the binding pocket; **C** view looking from the bilayer midplane up at the protein. Coloring is as follows: (for lipids) PIP_2_, red small spheres; POPA, blue small spheres; other lipids, gray lines; (for amino acids, space-filling) R, green; T, red; S, magenta; M, blue; A, yellow; V, violet; Y, orange; H, pink; L, cyan; G, silver. Other protein residues in gray ribbon style. The molecular surface was calculated based on van der Waals radii with a probe radius of 1.4 Å using the “Surf” module in VMD [72]. Water and ions are not displayed.

The molecular image of the largest cluster at 3μs is displayed in Fig 10. The hPLD2 inserted into the bilayer surface to form a small pothole-shaped binding pocket (Fig 10B), wherein PIP_2_ and POPA bind to their well-matched partners: while most of the PIP_2_ and POPA interact with hPLD2 at the surface of the bilayer, some PIP_2_ can be seen interacting with arginine residues (i.e., R287, R212, R281, R232, R217, and R226) well beyond the surface plane (Fig 10A and 10B). Here the specific interaction between protein residues and PIP_2_ results in 5 different kinds of major binding sites (i.e., three phosphate groups with different protonation states at P4 and P5 as listed in Table 2). These may play a key role in optimizing the binding-network geometry in the protein-membrane interface.

## 4. Discussion and conclusions

This study investigated the structural properties of human PLD2 (hPLD2) with a focus on interactions with PIP_2_. We first build a full-length model of hPLD2 combining template-based and *ab initio* modeling techniques in a two-step protocol, followed by molecular dynamics simulations of the protein in contact with a membrane.

The first step of the protocol modeled two different segments of hPLD2 (chains A and B) using the two identified templates by the HMM-based protocol implemented in HHpred server. Both templates and hPLD2 share good correspondence between secondary structural elements despite their extremely low sequence identity. The efficiency of such a template detection is mainly due to the use of HMM-profile-based protocols, which increase the sensitivity of distantly related homologs and have become a turning point in the molecular modeling field.

The second step utilized *ab initio* modeling to model *in situ* (onto chains A and B) the remainder of hPLD2 amino acids. This *in situ* modeling approach allowed us to take into account a possible interaction between chains A and B (modeled separately in the first step) by including the opportunity to define distance restraints. Mahankali *et al*. [69] assumed such interaction in their modeling protocol of hPLD2, in which they performed a protein-protein docking of both segments. Our coevolutionary analysis of hPLD2 sequence using the two most commonly used methods (EVFold and GREMLIN) did not identify any coevolved pairs indicating that most probably chains A and B do not interact with each other.

The preceding modeling protocol yielded a solution structure or one that might be obtained by x-ray crystallography, and thereby only provides indirect insight into how hPLD2 interacts with a cellular membrane. To remedy this deficiency MD simulations were generated on the modeled protein complexed to fluid phase membranes containing 10% and 1% PIP_2_/POPA (Table 1); the initial placement was based on the electrostatic potential maps (Fig 4) and docking analysis of PIP_2_ binding sites (Fig 5). As is evident in S5 Fig in S1 File, hPLD2 clearly interacts much more strongly with the 10% systems than those at 1% PIP_2_/POPA, which, as consistent with the electrostatics, are only loosely bound to the membrane. Nevertheless, trajectories of the 10% systems were not converged at 500 ns, and one of the replicates was extended to 3μs (Fig 6). The 2–3μs segment of this-trajectory was then used to characterize the interactions in molecular detail.

It is clear that PIP_2_ plays a primary role in hPLD2-membrane interactions and POPA promotes PIP_2_-mediated hPLD2-bilayer interactions. Furthermore, there is high residue specificity: 23 residues of the protein bind tightly to PIP_2_, as measured by the average number of contacts and residence times over 2–3 μs (Fig 8 and Table 2). In contrast, only 9 residues interact with heavy atoms of POPA with a similar count (S7 Fig and S1 Table in S1 File). The top 3 residues in terms of the average contact number for each lipid were all arginine; R142, R136, and R287 for PIP_2_ and R217, R226, and R232 for POPA. The top 3 for POPA also interact with PIP_2_. POPA has a simple binding mode: the primary binding atoms were on the phosphate group except for G220 and L221. In contrast, the PIP_2_ headgroup exhibited the complex binding patterns: arginine strongly binds to non-protonated P4 and/or P5, while other amino acids preferentially interact with the phosphate groups other than P4^np^ and P5^np^ (e.g., M1, T4, A3, and S288). The preferential binding sites for arginine were different depending on the locations of the residue (Table 2).

The results obtained indicate that not only the electrostatic potential generated by conserved clusters of basic residues and PIP_2_ headgroups (–4 at pH 7.0) can drive non-specific electrostatic attraction as proposed by McLaughlin and coworkers [73], but also the PIP_2_ headgroup, with three phosphate groups with different protonation states, induces specific bindings with well-matched their charged partners as recently shown for PIP_2_ monolayers [66]. Here these interactions led to the formation of clusters at the protein-membrane interface; the largest cluster was formed in the PH domain when not only PIP_2_ but also POPA linked lipids in the network (Figs 9 and 10).

The detailed description provided by all-atom molecular dynamics simulations comes with a high computational cost and hence is limited to only a few conditions (here the focus was on 10% PIP_2_ and POPA, and KCl). Furthermore, a detailed binding study (wherein the hPLD2 is systematically sampled a different distance from the membrane surface) was not feasible. There are other less computationally intensive approaches for investigating the binding of proteins to membranes. These include Poisson-Boltzmann calculations [73], simulation methods based on implicit membrane models [74], modifications of the lipid topology such as the HMMM [75], and coarse-grained models [76–78]. All accelerate sampling at some cost of physical realism compared to all atom-descriptions. Binding configurations obtained here could be used as the starting points for studies focusing on binding affinities between hPLD2 and bilayers based on the preceding methods.

Sampling is always a concern in MD simulations, especially for new systems such as this one. Ideally, numerous orientations near (but not at) the membrane surface would be simulated. These would bind, likely in different orientations, and eventually take on a small number of favorable poses. This is not computationally feasible for a large system such as this one. Here a single initial condition, based on the electrostatic map (S3 Fig in S1 File) and three replicates for each system (1% and 10% PIP2 and POPA) were generated. The 500 ns orientations of PLD2 were dramatically different for the 1% systems (S5 Fig in S1 File, left) and it was clear that there was insufficient computer power to adequately sample this concentration. In contrast, the three replicates for the 10% system yielded qualitatively similar binding orientations (S5 Fig in S1 File, right), though the number of heavy atom contacts differed (500 ns point of Fig 6). The trajectory of one of the replicates (R1) was extended to 3 μs; during this time, R1 developed contacts close to the average of R1-R3 at 500 ns (3 μs point of Fig 6). While the position and orientation of PLD2 do not change substantially between 1 and 3 μs (Fig 7, left), there was substantial diffusion and rearrangement of lipids (Fig 7). Hence, sampling for the 10% system appears to be adequate.

Unfortunately, there is presently little experimental data to directly test the proposed structure of hPLD2. There is no crystal structure. Docking studies in solution, as well as mutational studies, are most representative of PIP_2_ binding in its soluble form rather than the membrane-bound state simulated here. Predictions from the present structure are natural entry points for future experiments such as cys accessibility analysis to determine if, for example, a certain loop or region is indeed exposed to the solvent instead of being buried within the fold of the protein. Mutagenesis analysis of conserved residues that are exposed would help identifying key residues for PLD2 function, subcellular localization or interaction with other proteins, especially when combined with Yeast Two Hybrid and pull-down assays. In vitro lipid binding experiments could be also used to validate if the predicted residues in this work bind PIP2. This type of experiment has been successfully used in other systems such as the Dopamine Transporter [79].

In summary, this study developed a structural model of hPLD2 and characterized interactions between the protein and PIP_2_-containing membranes using first a protocol combining template-based and *ab initio* molecular modeling techniques and then microsecond molecular dynamics simulations in the presence of a complex membrane. The model provides a starting point for understanding how this critical enzyme mediates numerous physiological processes via dynamical interactions with membranes, and how it might be regulated by membrane composition.

## Supporting information

S1 File (PDF)
