# Supplementary material for: PLD2–PI(4,5)P2 interactions in fluid phase membranes: Structural modeling and molecular dynamics simulations
Source: PLoS One. 2020 Jul 20;15(7):e0236201. doi: 10.1371/journal.pone.0236201 (PMC7371163; doi:10.1371/journal.pone.0236201)
Supplement: S1 File — (PDF) [file pone.0236201.s001.pdf]

SUPPORTING INFORMATION

PLD2–PI(4,5)P_2 Interactions in Fluid Phase Membranes: Structural Modeling and Molecular Dynamics Simulations

Kyungreem Han[1,#a], Richard W. Pastor[1], and Cristina Fenollar–Ferrer[2-4,*]

[1]Laboratory of Computational Biology, National Heart, Lung and Blood Institute, National Institutes of Health, Bethesda, MD 20892, USA

[2]Laboratory of Molecular & Cellular Neurobiology, National Institute of Mental Health, National Institutes of Health, Bethesda, MD 20892, USA

[3]Laboratory of Molecular Genetics, National Institute on Deafness and other Communication Disorders, Bethesda, MD 20892, USA

[4]Molecular Biology and Genetics Section, National Institute on Deafness and other Communication Disorders, MD 20892, USA

[#a]Current Address: Center for Neuroscience, Brain Science Institute, Korea Institute of Science and Technology, Seoul, 02792, Korea

*Corresponding author

E-mail: cristina.fenollarferrer@nih.gov (CFF)

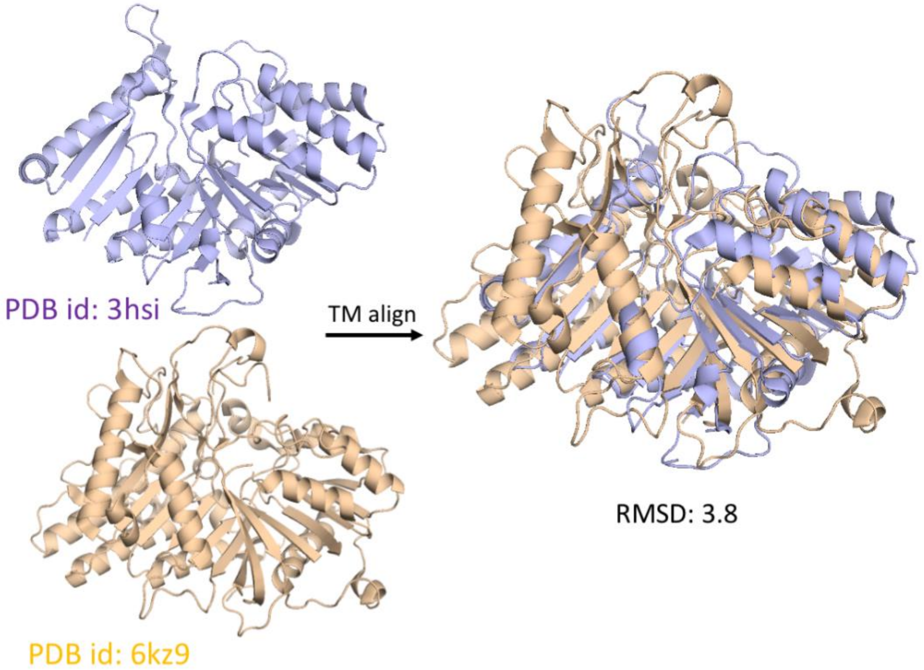

S1 Fig. Structural superimposition of the X-ray structures of Phosphatidylserine synthase from Haemophilus influenzae (PDB id: 3hsi), used as a template in this study, and Phospholipase D alpha 1 from Arabidopsis thaliana (PDB id: 6kz9). The segments structurally superimposed constitute the core fold of these proteins and are those identified in HHpred with similar fold to the C-ter of hPLD2. The core fold is common in both structures with an RMSD of 3.8 A.

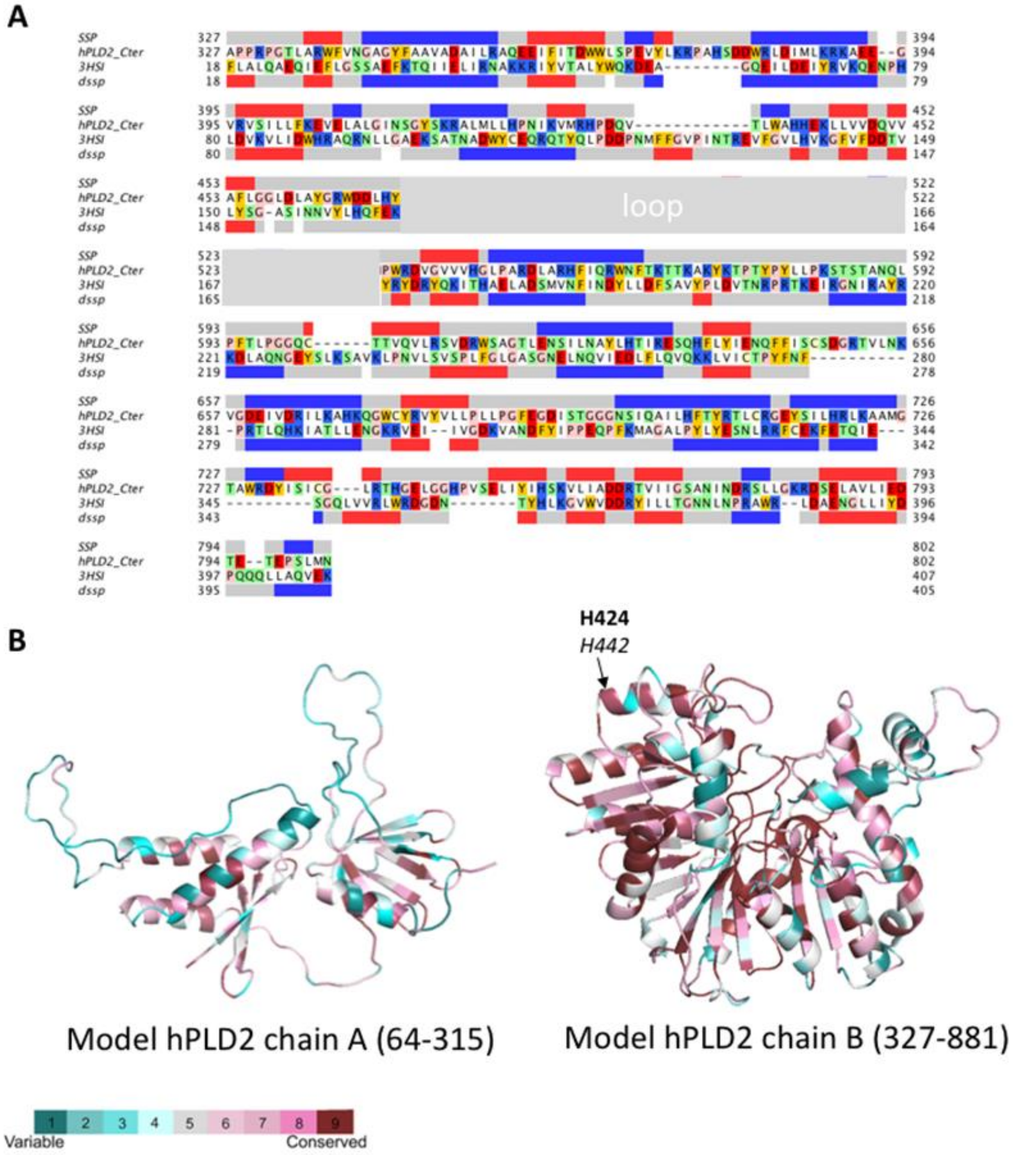

S2 Fig. (A) The initial alignment obtained with HHpred between the C-ter of hPLD2 and template 3hsi. The secondary structure of the template and that predicted for hPLD2 are indicated as gray (coil), blue (helix) and red (strand) bars at the bottom and top of the corresponding sequence.
(B) The models obtained for the N- and C-ter of hPLD2 in ribbon representation and colored by residue conservation. Residue H424 (equivalent to H442 in hPLD1) is indicated by an arrow.

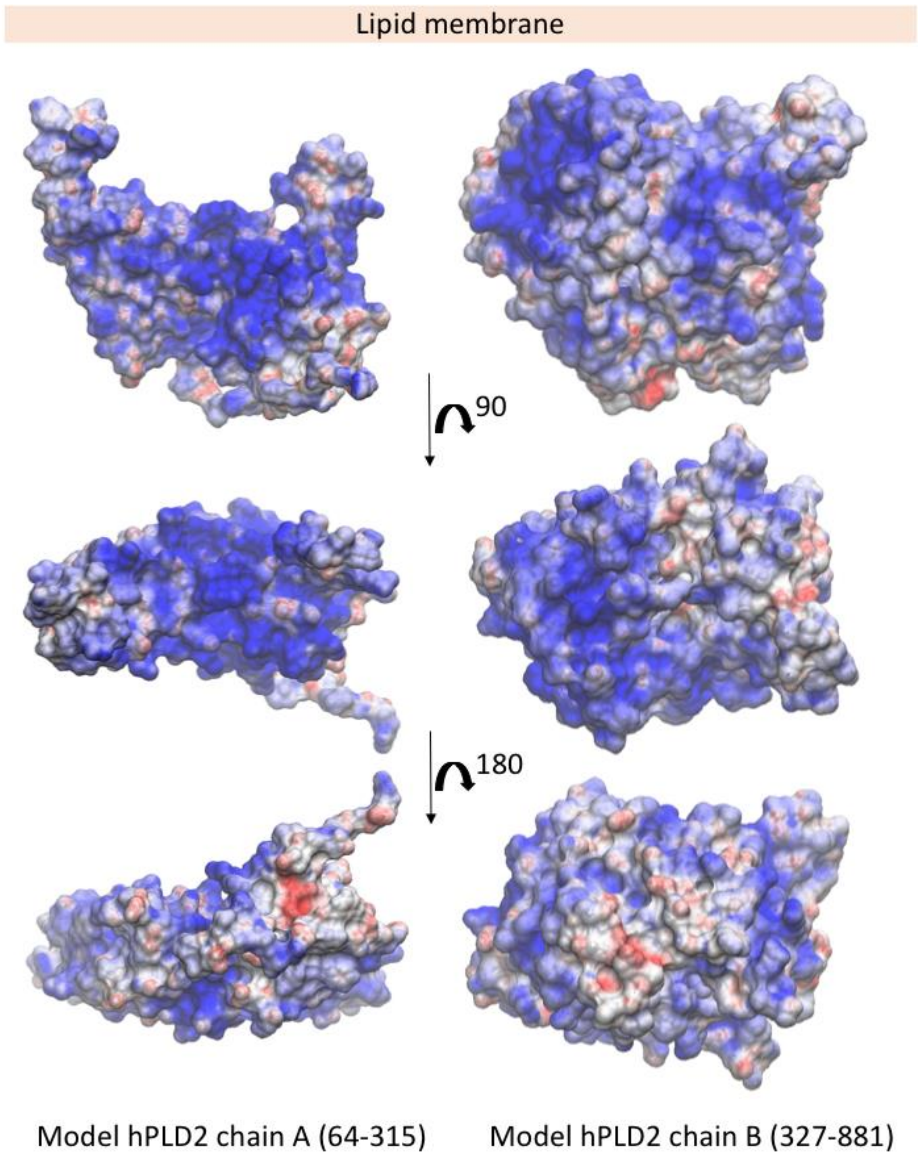

S3 Fig. Electrostatic potential mapped on the surface of N- and C-terminal modeled segments of hPLD2 shown in side, top, and bottom views. The membrane surface is depicted as a tan rectangular slab for the side view only.

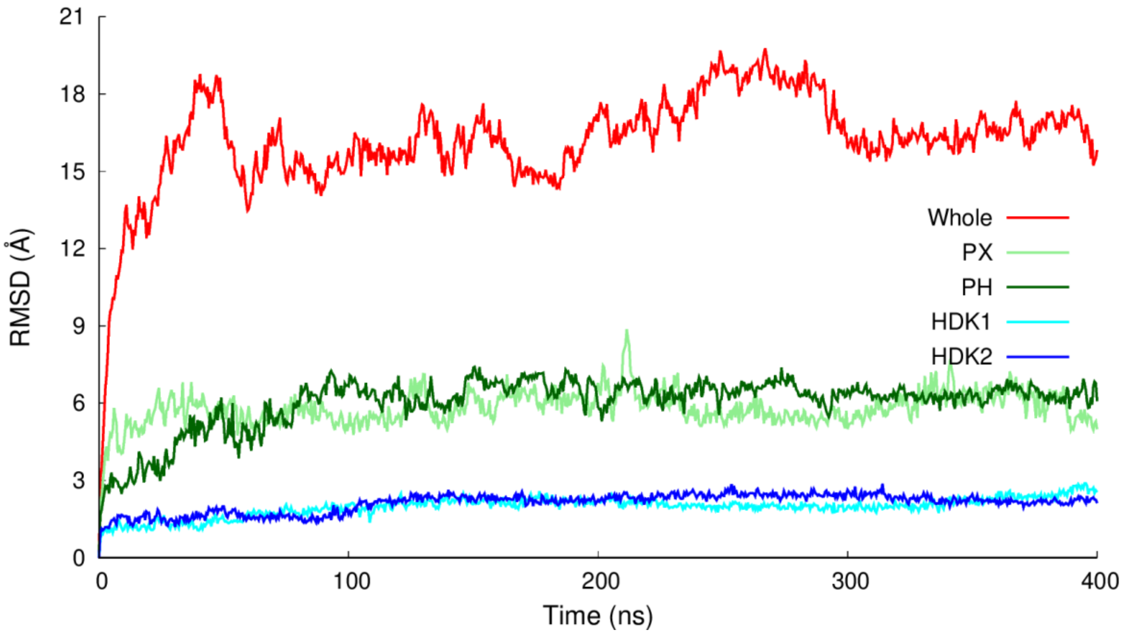

S4 Fig. Time evolution of the atom positional root-mean-square deviation (RMSD) of C_α of the entire protein (indicated as 'Whole') and domains (i.e., PX, PH, HDK1, and HDK2).

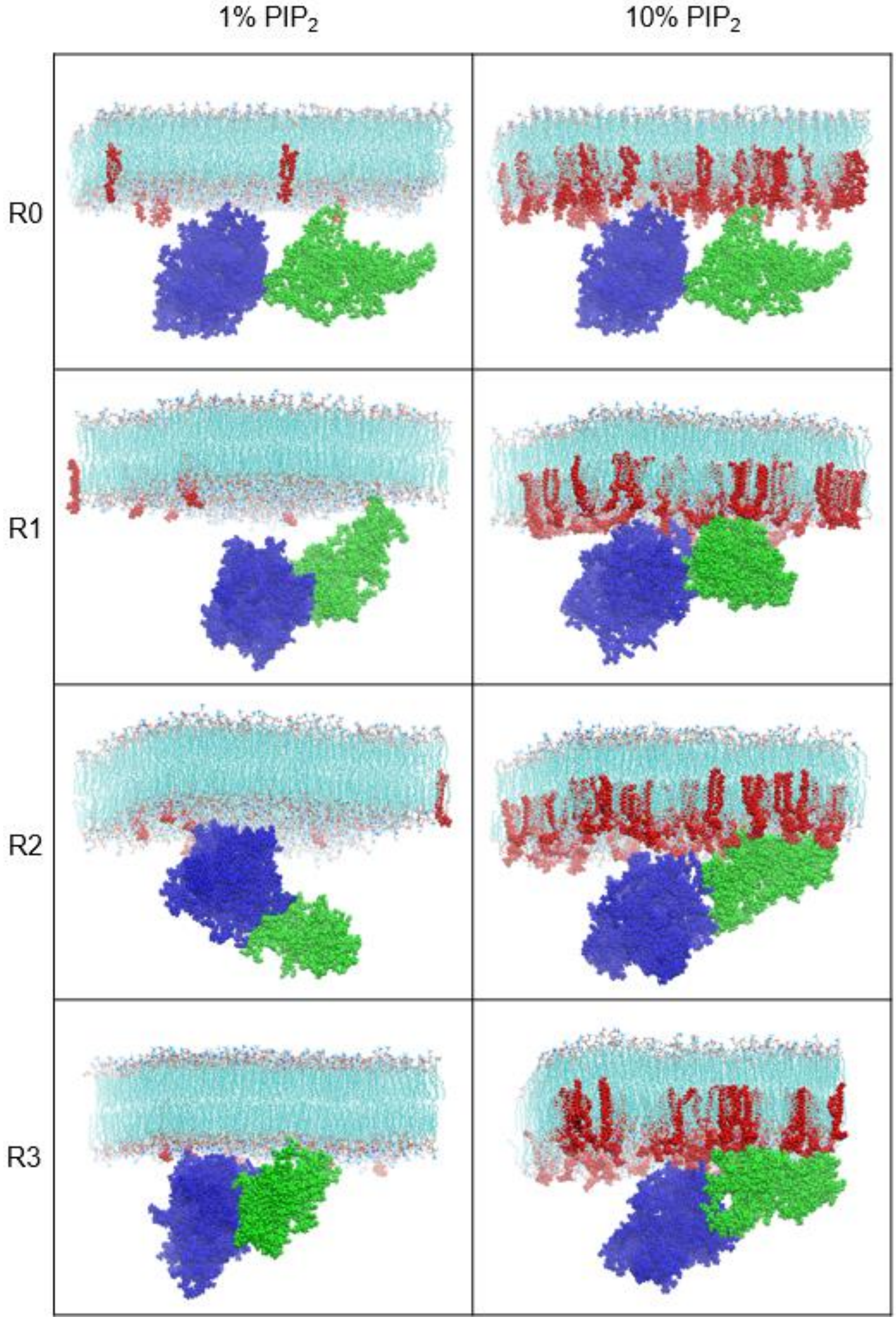

S5 Fig. 500ns snapshots of (left) 1% PIP_2 and (right) 10% PIP_2 bilayer systems. R1, R2, and R3 denote the three replicas of each system and R0 the initial configuration of the replicas. Coloring is as follows: PIP_2, red; N-term (PLD2 residues from 1 to 325), green; C-term (326 to 933), blue. Water and ions are not displayed. The starting position of PLD2 in the six systems was identical for all six systems, as were the positions of the lipid atoms for the two concentrations of PIP_2. The initial velocities were different for each replicate.

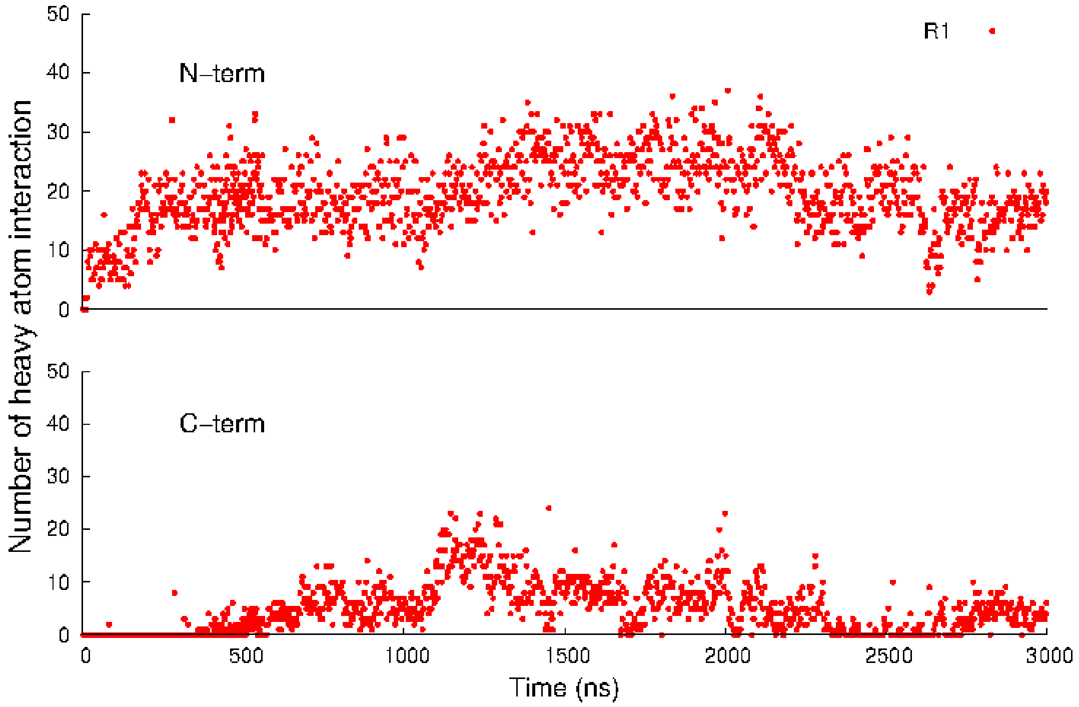

S6 Fig. 3μs time profiles of PLD2-POPA interactions of one of the 10% PIP_2 systems (R1). The interaction indicates the numbers of contacts between the heavy atoms of PLD2 and POPA within 3.5 Å. It was calculated for two groups of PLD2 separately: N-term in the first row and C-term in the second row.

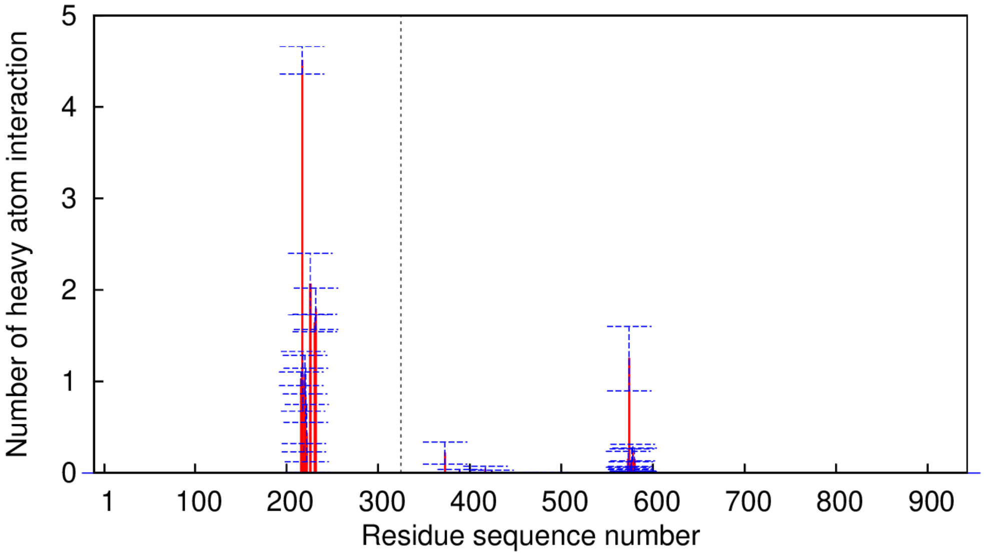

S7 Fig. The interactions between POPA and PLD2 residues. The interaction indicates the numbers of contacts between the heavy atoms of POPA and each of PLD2 residues within 3.5 Å. Data were obtained from the last 1μs trajectories of the replica R1 of the 10 % system, dividing into 5 blocks of 200ns to estimate the average and standard error (SE). The vertical dotted line indicates the border between N-term and C-term.

S1 Table. POPA binding sites.

Ranking	Residue	[b]Domain	[a]Average contacts with POPA (mean ±S.E.)		%Binding lifetime (Number of POPA partners)	Binding site ratio (%)	
						$P1^p$	other
1	R217	N-PH	4.508 ±0.150		100.0 (2)	76.7	23.3
2	R226	N-PH	2.063 ±0.336		83.1 (2)	97.9	2.1
3	R232	N-PH	1.793 ±0.224		79.0 (1)	100.0	0.0
4	Y231	N-PH	1.639 ±0.093		88.0 (1)	100.0	0.0
5	K574	C	1.248 ±0.354		57.8 (3)	96.5	3.5
6	G220	N-PH	1.072 ±0.211		63.9 (3)	31.9	68.1
7	H216	N-PH	1.027 ±0.074		81.7 (1)	100.0	0.0
8	V218	N-PH	1.000 ±0.326		59.3 (3)	66.9	33.1
9	L221	N-PH	0.848 ±0.297		55.4 (3)	50.3	49.7

[a]All residues with average contacts > ~1 are displayed. [b]N and C denote 'N-term' and 'C-term' and PH the pleckstrin homology domain. P1 indicates heavy atoms of the phosphate group attached to C1 and the superscript 'p' denotes 'protonated' and 'other' the binding sites of POPA other than P1. %Binding lifetime denotes the fraction of time that a certain residue bound to neighboring POPA which are identified in parentheses. Data were obtained from the last 1μs trajectories of the replica R1 of the 10% system.
